# Continuous Estimation of Annual Committed Effective Dose of Radioactive Cesium by Market Basket Study in Japan from 2013 to 2019 after Fukushima Daiichi Nuclear Power Plant Accident

**DOI:** 10.14252/foodsafetyfscj.D-20-00017

**Published:** 2020-12-25

**Authors:** Hiromi Nabeshi, Tomoaki Tsutsumi, Masataka Imamura, Yoshinori Uekusa, Akiko Hachisuka, Rieko Matsuda, Reiko Teshima, Hiroshi Akiyama

**Affiliations:** 1 Division of Foods, National Institute of Health Sciences, Kanagawa, Japan 3-25-26 Tonomachi, Kawasaki-ku, Kawasaki, Kanagawa 210-9501, Japan; 2 Division of Natural Medicines, Faculty of Pharmacy, Keio University, Tokyo, Japan 1-5-30 Shibakoen, Minato-ku, Tokyo 105-8512, Japan; 3 Division of Biochemistry, National Institute of Health Sciences, Kanagawa, Japan 3-25-26 Tonomachi, Kawasaki-ku, Kawasaki, Kanagawa 210-9501, Japan; 4 Division of Food Safety Information, National Institute of Health Sciences, Kanagawa, Japan 3-25-26 Tonomachi, Kawasaki-ku, Kawasaki, Kanagawa 210-9501, Japan; 5 Faculty of Veterinary Medicine, Okayama University of Science, Ehime, Japan 1-3 Ikoinooka, Imabari, Ehime 794-8555, Japan

**Keywords:** Key word annual committed effective dose, daily intake, Fukushima Daiichi Nuclear Power Plant accident, market basket, radioactive cesium

## Abstract

Radionuclide contamination in foods has been a great concern after the Fukushima Daiichi Nuclear Power Plant (FDNPP) accident. To estimate time trends of daily intake and annual committed effective dose of radionuclides after the accident, radioactive cesium (r-Cs; ^134^Cs and ^137^Cs) and potassium-40 (^40^K) in market basket (MB) samples prepared at 6-month intervals in periods from September 2013 to March 2019 in 15 regions of Japan were analyzed using γ-ray spectrometry. The annual committed effective dose of r-Cs, calculated at non-detected radionuclide levels assumed to be half the limit of detection (LOD), appeared to decrease gradually in 11 regions close to the FDNPP that were more likely to be affected by the accident. Differences in doses among the 15 regions were large just after the accident, but gradually decreased. In particular, ^134^Cs has not been detected in any MB sample in any region since September 2018, and annual committed effective dose from ^134^Cs in all regions was mostly constant at around 0.3 μSv/year (given the respective LODs). The maximum annual committed effective dose of r-Cs in this study was decreased from 2.7 μSv/year in September 2013 to 1.0 μSv/year in March 2019. In contrast, the range of annual committed effective dose of ^40^K varied from approximately 150 to 200 μSv/year during that time frame and did not change much throughout the period of this study. Although annual committed effective doses of r-Cs in regions close to the FDNPP appeared to be higher than in regions far from the FDNPP, doses in all regions are remaining at a much lower levels than the intervention exemption level, 1 mSv/year, in foods in Japan.

## 1. Introduction

The Fukushima Daiichi Nuclear Power Plant (FDNPP) accident happened on March 2011 due to the tsunami triggered by the Tohoku earthquake. After the accident, several different types of radionuclides were released from the FDNPP into the surrounding environment. According to a report released by the Nuclear and Industrial Safety Agency (NISA)^1^^)^, these radionuclides included xenon-133 (^133^Xe; approximately 1.1 × 10^19^ Bq), iodine-131 (^131^I; approximately 1.6 × 10^17^ Bq), cesium-134 (^134^Cs; approximately 1.8 × 10^16^ Bq), and cesium-137 (^137^Cs; approximately 1.5 × 10^16^ Bq). Although the released total quantities of ^133^Xe, ^131^I, and ^132^Te were substantially large for a short time after the accident, the half-lives of these radionuclides are very short (less than 10 days)^2^^)^. Therefore, one year after the FDNPP accident, the largest remaining amounts in the environment are thought to be mainly ^134^Cs and ^137^Cs. However, since large quantities of radionuclides were released and remain in the environment, many foodstuffs were directly or indirectly contaminated with radionuclides.

Responding to this situation, the government of Japan immediately established provisional regulatory values and started to regulate the distribution of foods contaminated with radionuclide amounts that exceeded those values to ensure food safety^3^^)^. In April 2012, new standard limits were established for foods (10, 50, and 100 Bq/kg for drinking water, milk and infant foods, and general foods, respectively, as the concentration of radioactive cesium [r-Cs; the sum of ^134^Cs and ^137^Cs]) by the Ministry of Health, Labour and Welfare (MHLW) based on risk assessments by the Food Safety Commission in Japan^4^^)^. These standard limits were set to avoid exceeding the intervention exemption level of the Codex Alimentarius Commission (CODEX), 1 mSv/year, a level considered as safe for the public.

Monitoring the level of r-Cs in foods has continued by local governments to avoid distributing foods contaminated with r-Cs levels higher than the standard limits. To confirm the validity of the risk management, it is important to understand actual situations about r-Cs internal exposure dose from foods. However, because r-Cs concentration in foods is probably changed by processing or cooking^5^^,^^6^^,^^7^^,^^8^^,^^9^^)^, concentrations of radionuclides in uncooked foods, which are the main targets of monitoring, are not necessarily directly reflected to dietary intake. Therefore, to estimate more realistic dietary intake of r-Cs, it is necessary to consider the possibility of r-Cs concentration change by processing or cooking.

To estimate the dietary intake of radionuclides, especially r-Cs, many surveys have been conducted after the FDNPP accident^10^^,^^11^^,^^12^^,^^13^^,^^14^^,^^15^^)^, and a review of internal exposure dose based on results from these surveys was published^16^^)^. We also reported on daily intake and annual committed effective dose of r-Cs using a market basket (MB) study design^17^^,^^18^^)^. Previous studies including our survey suggested that annual committed effective dose of r-Cs in Japan was sufficiently lower than the intervention exemption level, 1 mSv/year. The time periods in these surveys were mainly within three years after the FDNPP accident, and the dietary intake of r-Cs estimated at the end of these short periods might not exactly reflect the influence of r-Cs contamination in foods because some of the foods contaminated by r-Cs released by the accident, such as processed foods, might have not yet been distributed. Long-term transitional changes in dietary intake of r-Cs have not yet been reported. To consider these issues, continuous surveys across wide areas have been required. In addition, it would be important to monitor daily intake and annual committed effective dose of r-Cs because those reflect r-Cs concentrations in commercial foods in Japan. Therefore, continuous surveys of dietary intake of r-Cs are necessary to evaluate the effects of food regulations resulting from standard limits on r-Cs.

In this study, to evaluate the time trends of dietary intake of r-Cs, particularly concerned radionuclides after the FDNPP accident, we estimated daily intake and annual committed effective dose of r-Cs by analyzing MB samples derived from 15 regions in Japan during the periods from September 2013 to March 2019. For comparison, we also estimated daily intake and annual committed effective dose of potassium-40 (^40^K), which is a representative natural radionuclide. Our findings were then compared to the results of surveys reported before the FDNPP accident.

## 2. Materials and Methods

### 2.1 Materials and Methods

**Fig. 1.**
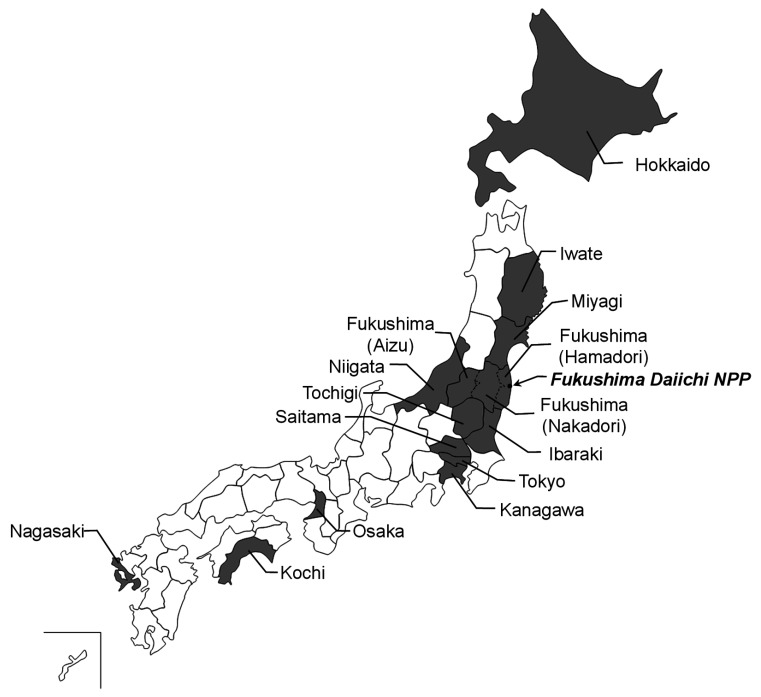
Fifteen regions in Japan where MB samples were prepared. Filled 15 regions on the map are subject regions of r-Cs intake survey. Filled circle indicates location of the FDNPP.

MB samples were prepared twice a year, in March periods (Mar-) and in September periods (Sep-), from September 2013 to March 2019. Based on the 2008-2010 (study period from Sep-2013 to Mar-2016) and the 2011-2013 (study period from Sep-2016 to Mar-2019) results from the National Health and Nutrition Examination Survey (≥1 year of age) in Japan, types of foods and daily food consumption data used in the present study were decided by each region. The foods were purchased from local supermarkets in each of the 15 regions (three areas in Fukushima [Nakadori, Hamadori, Aizu], Iwate, Miyagi, Tochigi, Ibaraki, Saitama, Tokyo, Kanagawa, Niigata, Hokkaido, Osaka, Kochi, and Nagasaki, as shown in Fig. 1). To estimate annual committed effective dose in the MB sample when locally produced foods were consumed preferentially, locally produced foods were selected as ingredients of MB samples, especially the fresh foods category that included fruits, vegetables, and fish, to the extent possible. If unable to purchase locally-produced foods, neighboring area-produced foods and domestic foods were preferentially selected. All foods purchased were classified into the following 13 categories summarized in Table 1; category 1, rice and rice products (3 kinds of food); category 2, cereals, potatoes, and nuts (29 kinds of food); category 3, sugar and confectioners (12 kinds of food); category 4, oil and fats (5-6 kinds of food); category 5, beans and their products (10-11 kinds of food); category 6, fruits (11 kinds of food); category 7, colored vegetables (9 kinds of food); category 8, vegetables, mushrooms, and seaweeds (15 kinds of food); category 9, alcohol and beverages (10 kinds of food); category 10, fishes and shellfishes (24-25 kinds of food); category 11, meats and eggs (13-14 kinds of food); category 12, milk and milk products (7 kinds of food); and category 13, seasonings and spices (12 kinds of food). Tap water was used as drinking water (category 14). These categories were used for estimation of dietary intake of contaminants such as dioxins^19^^)^. Foods were weighted in proportion to the amount of daily consumption by each region. Foods were washed, peeled, and cooked easily such as by boiling and stir-frying without oil and seasoning, according to Japanese eating habits. Foods were then mixed well in a blender without adding water, by each food category and each region. All the prepared MB samples (total 210; 15 regions × 14 food categories) were stored below -20°C until the analysis.

**Table 1.**
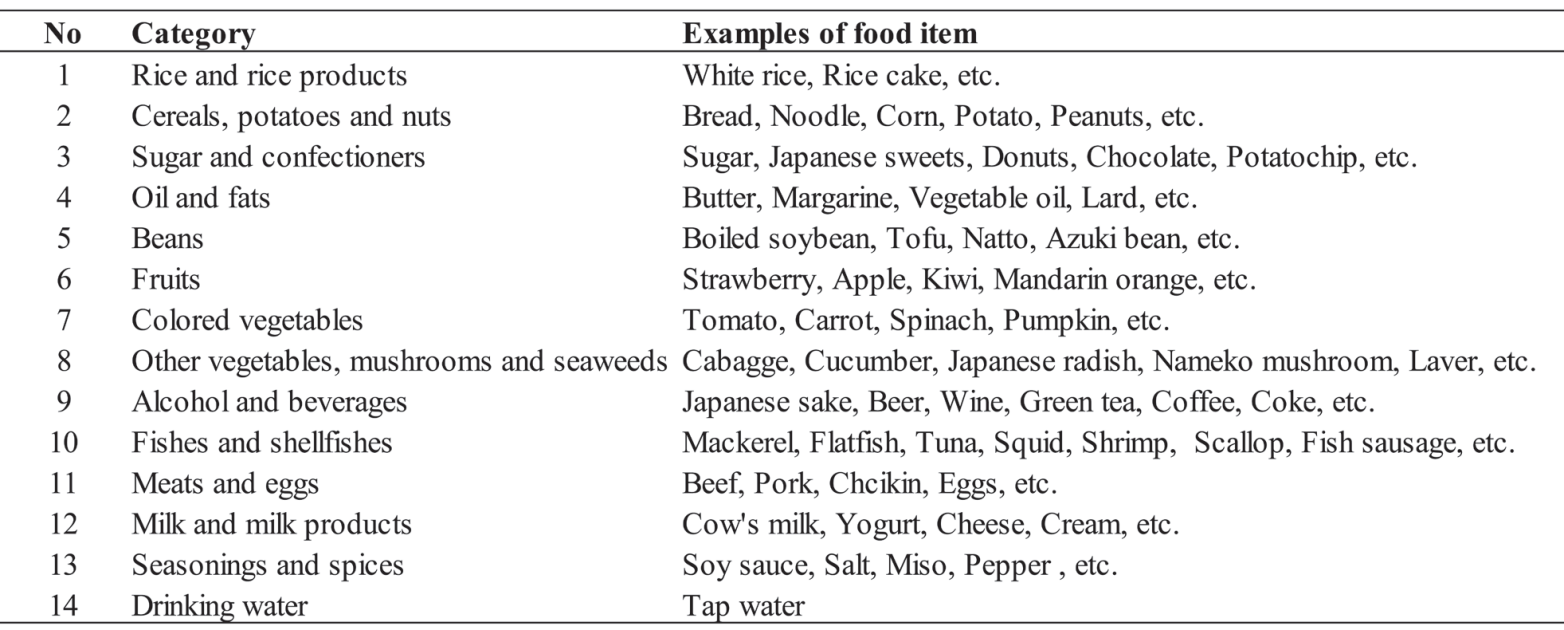
Classification of food categories in MB samples

### 2.2 Determination of ^134^Cs, ^137^Cs, and ^40^K

Radioactive Cs (r-Cs; ^134^Cs, and ^137^Cs) and ^40^K in all prepared MB samples (15 regions in 12 periods) was measured by a high-purity Germanium (HPGe) γ-spectrometer (GC4019; Canberra, Meriden, CT, USA). Each sample was filled into a 2 L Marinelli container and measured for 22 hrs. Analytical software (Gamma Explorer; Canberra) was used to calculate r-Cs and ^40^K concentrations from the HPGe γ-spectrometer data. Matrix was set to “water”, and self-absorption was corrected. Results were corrected for the background (from a measurement conducted for 48 hrs), the sum effect, and the attenuation (reference date was the date of sample preparation). Because ^134^Cs emits several different energy gamma-rays, concentration of ^134^Cs was calculated using load average concentration from peaks of 475.4, 563.3, 569.3, 604.7, 795.8, 801.8, 1038.5, 1167.9, and 1365.1 keV^20^^)^. Concentrations of ^137^Cs and ^40^K were calculated using counts from peaks of 661.6 keV and 1460.75 keV, respectively. The detector was regularly calibrated using a gamma ray reference source purchased from the Japan Radioisotope Association (Tokyo, Japan). Peak area was calculated by an extension method or function adjustment method, depending on the peak shape. The concentration of r-Cs represents the sum of concentration of ^134^Cs and ^137^Cs. Lower limits of detection (LOD) of ^134^Cs, ^137^Cs, and ^40^K were 0.028~0.098, 0.029~0.088, and 0.22~1.5 Bq/kg, respectively. LOD is affected by various factors such as the air dose in the measurement environment and the background in each MB sample. Because the measurement environment changed in August 2017, LODs of r-Cs and ^40^K in several food categories after Sep-2017 tended to be somewhat lower than those before Mar-2017.

### 2.3 Calculation of Daily Intake and Annual Committed Effective Dose of r-Cs and ^40^K

The daily intake of each radioactive nuclide in each category was determined by multiplying the concentration of each radioactive nuclide by food consumption. The daily intake of each radioactive nuclide in each region was calculated as the sum of all categories (categories 1 to 14). If the concentration of each radioactive nuclide was lower than each LOD (=ND), half value of LOD (1/2LOD) was used as the concentration for calculation of daily intake. According to the recommended method by Global Environmental Monitoring System (GEMS) for handling data on foods with low levels of contamination, in food categories with concentrations lower than LOD^21^^)^, if the data set contains more than 60% of data that is higher than LOD, all data lower than LOD are calculated as 1/2LOD. Taking data continuity into account, annual committed effective dose of r-Cs was calculated by the same method used before 2013, although detection rates for r-Cs, especially ^134^Cs, were below 60% since 2013. The daily intake per person in each region was calculated using the following equation:

$$\sum_{i=1}^{14}\left(Cki\;\times \;Mi\;\right)$$

where *k* is the radionuclide, D*k* is the daily intake of the radionuclide *k* per person, *i* is the individual food category, C*ki* (Bq/kg) is concentration of radionuclide *k* in food category *i*, and M*i* (kg/day) is the daily consumption of food category *i*.

The annual committed effective doses of each radionuclide were calculated using the following equation, assuming that the daily intake of radioactive nuclides was constant throughout the year:

E*k***(**μSv/person/year**)** =
D*k* (Bq/person/day) × A*k* (μSv/Bq) × 365
(days)

where E*k* is the annual committed effective dose of radionuclide *k*, and A*k* is committed effective coefficients for radionuclide *k* by ingestion. The committed effective coefficients for adults obtained were 1.9 × 10^−2^ μSv/Bq for ^134^Cs, 1.3 × 10^−2^ μSv/Bq for ^137^Cs, and 6.2 × 10^−3^ μSv/Bq for ^40^K^22^^)^.

## 3. Results and Discussion

### 3.1 Detection Rates and Concentrations of r-Cs and ^40^K in MB Samples

**Fig. 2.**
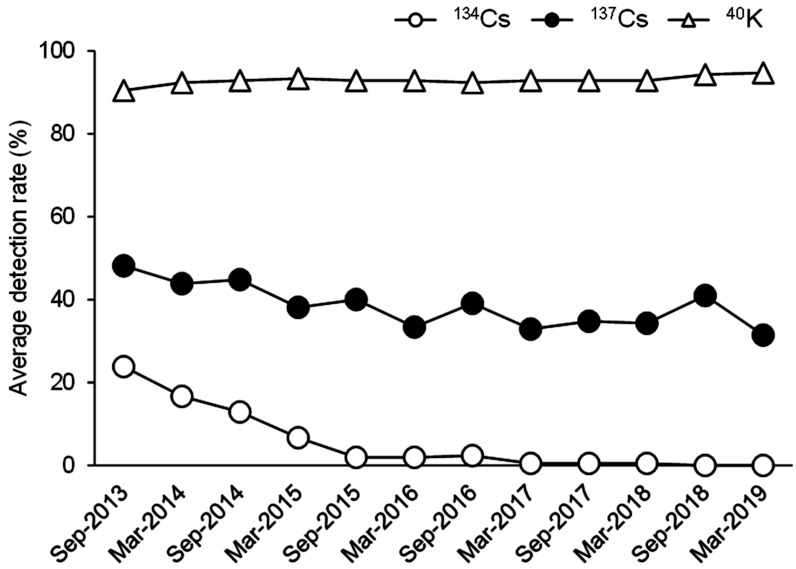
Time trends of average detection rates for ^134^Cs, ^137^Cs, and ^40^K. Opened circles, filled circles, and opened triangles indicate average detection rates for ^134^Cs, ^137^Cs, and ^40^K, respectively.

We investigated the time trends of detection rates and concentrations of r-Cs and ^40^K in MB samples. From the total of 210 MB samples (15 regions × 14 food categories) analyzed in each period, the average detection rates for ^134^Cs, ^137^Cs, and ^40^K are shown in Fig. 2. The detection rate for ^134^Cs decreased substantially from 24% to 0% during the 5.5-year duration of this study. The detection rate for ^137^Cs also decreased from 48% to 31%, although this decrease was slower than that for ^134^Cs. The difference in the rate decrease between ^134^Cs and ^137^Cs was thought to be due to a difference in their half-lives. The physical half-life of ^134^Cs, approximately 2 years, is markedly shorter than that of ^137^Cs, which is approximately 30 years^2^^)^. Therefore, according to an example calculation based on physical half-life, the amount of ^134^Cs residue in 2013 and 2019 is estimated to be approximately 50% and 6%, respectively, compared with the total amount of released ^134^Cs at the time of the FNDPP accident, whereas the amount of ^137^Cs residue in 2013 and 2019 is estimated to be approximately 95% and 83%, respectively. Thus, the difference between amount of residue for ^134^Cs and ^137^Cs is getting larger over time and is related to time trends of the detection rates for ^134^Cs and ^137^Cs.

**Fig. 3.**
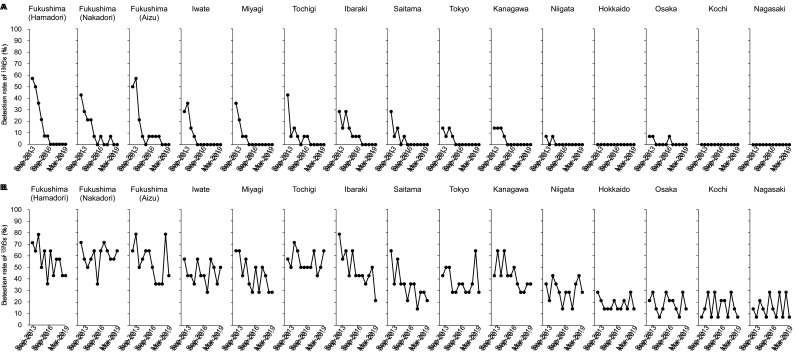
Time trends of detection rate for r-Cs in each region. Detection rate for ^134^Cs and ^137^Cs in each region are shown in A and B, respectively. Data shown according to the time of study.

Fig. 3 shows time trends of the detection rate for r-Cs in each region. Regions close to the FDNPP including Fukushima (Nakadori, Hamadori, Aizu), Iwate, Miyagi, Tochigi, Ibaraki, Saitama, Tokyo, Kanagawa, and Niigata were defined as near-FDNPP regions, and regions far from the FDNPP including Hokkaido, Osaka, Kochi, and Nagasaki were defined as far-FDNPP regions. The results indicated that there were regional differences in the detection rates for r-Cs and associated time trends. The detection of ^134^Cs in the near-FDNPP regions except Niigata gradually decreased, it was not detected in any region after Sep-2018, and it was never detected in the far-FDNPP regions except Osaka during the study period (Fig. 3A). The detection rate for ^137^Cs appeared to be higher in the near-FDNPP regions compared with the far-FDNPP regions, which were constantly low (approximately 30% lower) during the 5.5-year duration of this study (Fig. 3B). The detection rate for ^137^Cs in Fukushima (Hamadori), Miyagi, Ibaraki, Saitama, and Kanagawa appeared to gradually decrease. Meanwhile, the detection rates for ^137^Cs in the other near-FDNPP regions did not appear to decrease until March 2019.

**Fig. 4.**
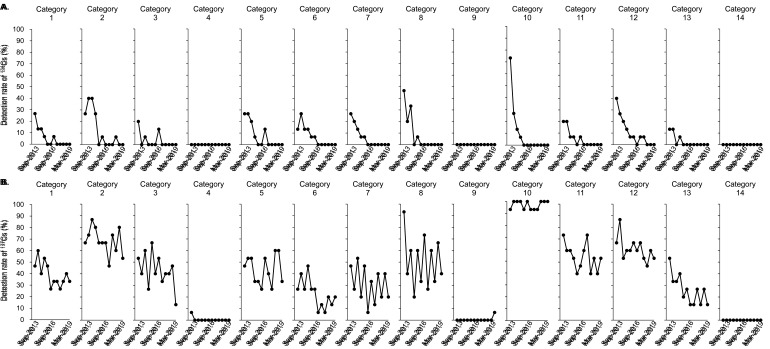
Time trends of detection rate for r-Cs in each food category. Detection rate for ^134^Cs and ^137^Cs in each food category is shown in A and B, respectively. Data are shown according to the time of study.

**Fig. S1.**
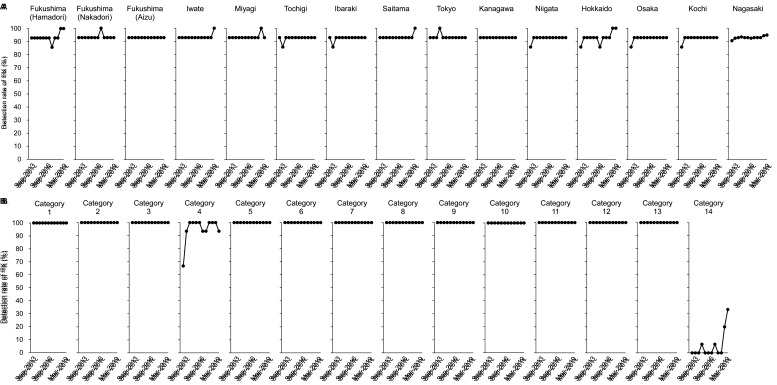
Time trends of detection rates for 40K in each region and each food category. Detection rates for 40K in each region and each food category are shown in A and B, respectively. In each region and each food category, data are shown according to the time of study.

Time trends for the detection rate for r-Cs in each food category during this study appeared to follow two patterns: one decreased gradually, and the other did not change (Fig. 4). The detection rates for ^134^Cs in all categories except 4, 9, and 14 decreased rapidly compared with ^137^Cs, which was likely a result of the difference in physical half-life between ^134^Cs and ^137^Cs. The presence of ^134^Cs was detected only in categories 2 and 12 since Sep-2016, and was not detected in any category since Sep-2018 (Fig. 4A). The detection rate for ^137^Cs in categories 1, 3, 6, 11, 12, and 13 appeared to decrease gradually during the study, while the detection rate in categories 2, 5, 7, 8, and 10 did not appear to decrease (Fig. 4B). The detection rate for ^137^Cs in categories 2, 10, 11, and 12 was over 50% in most cases throughout this study, especially in category 10, which consisted of fishes and shellfishes and had a detection rate of more than 90%. A similar tendency was reported in a study conducted by Sugiyama et al^23^^)^ before the FDNPP accident. This suggests that although the influence of global fallout and the Chernobyl nuclear accident remained in all food categories, their influence on category 10 was severer than other food categories. According to calculations based on the physical half-life of ^137^Cs, approximately 45% of total ^137^Cs released by the Chernobyl nuclear accident in 1986 is assumed to still remain in the environment in 2019. Because the detection rate in category 10 was high and the regional difference was small, we considered that category 10 in our study contained ^137^Cs that was derived from global fallout and the Chernobyl nuclear accident as well as from the FDNPP accident. Although the detection rate in category 10 remained high during this study, the average concentration of r-Cs in category 10 decreased year by year (data not shown). Conversely, r-Cs in categories 4, 9, and 14 was never detected except for ^137^Cs, which was detected only once in categories 4 and 9. Category 4 consisted of oils and fats. Since Cs are present as hydrophilic compounds, it is difficult to transfer from ingredients to oil and fats^24^^)^. In addition, oils are often made from imported ingredients. Therefore, r-Cs was unlikely to be detected in category 4 in almost all cases. Categories 9 and 14 are beverages including alcohol and tap water, respectively. Since tap water and water for food manufacturing are strictly managed by Water Works and have the same standard limit as drinking water (10 Bq/kg), r-Cs were also unlikely to be detected in categories 9 and 14 in almost all cases. On the other hand, average detection rates for ^40^K in all regions were always over 80% throughout the study period (Fig. 2). There was little difference in detection rates for ^40^K among regions, and detection rates for ^40^K in food categories except categories 4 (Sep-2013) and 14 were nearly 100% (Figs. S1A and S1B).

**Fig. 5.**
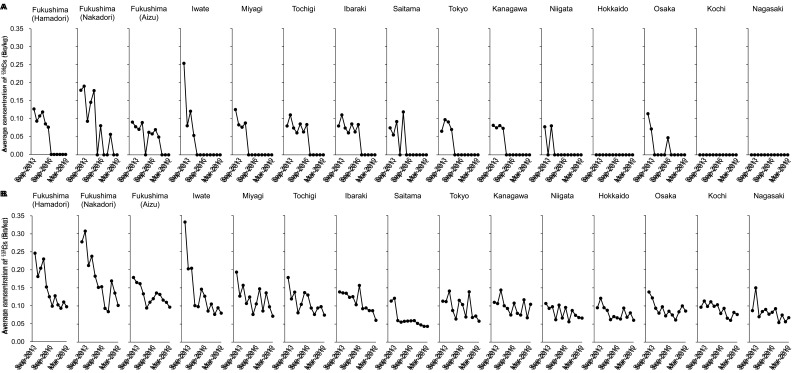
Time trends of average concentrations of r-Cs in each region. Average concentrations of ^134^Cs and ^137^Cs in each region are shown in A and B, respectively. In each region, data are shown according to the time of study. Average concentrations were calculated using only detected data. When detection rates of radionuclides in all samples were zero in each region, the data were expressed as zero.

**Fig. S2.**
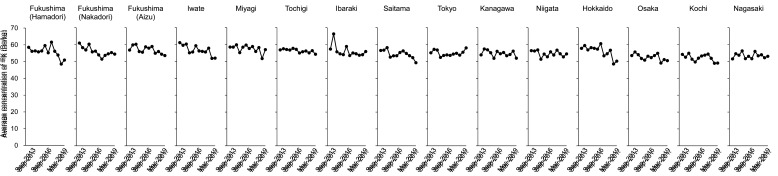
Time trends of average concentrations of 40K in each region. Data are shown according to the time of study. Average concentrations were calculated using only detected data. When detection rates for radionuclides in all samples were zero in each region and each food category, the data were expressed as zero.

Regional differences in average concentrations of r-Cs were apparent, but their corresponding time trends were mostly the same in all regions (Fig. 5). Relatively high concentrations were found in Fukushima (Nakadori) and Iwate until Sep-2015 and Sep-2014, respectively (Fig. 5A). Average concentrations of ^134^Cs in Fukushima (Nakadori) were 0.18, 0.19, 0.093, 0.15 and 0.18 Bq/kg in Sep-2013, Mar-2014, Sep-2014, Mar-2015 and Sep-2015, respectively and those in Iwate were 0.25, 0.080 and 0.12 Bq/kg in Sep-2013, Mar-2014 and Sep-2014, respectively. After that, the concentrations decreased to less than the LODs along with other regions where ^134^Cs was detected. Average concentrations of ^137^Cs tended to decrease gradually in all regions (Fig. 5B). Initially, concentrations of ^137^Cs in Fukushima, Iwate, Miyagi, and Tochigi appeared to be higher than in other regions including the far-FDNPP. However, the difference of average concentration of ^137^Cs between these two regions became very small in Mar-2019. On the other hand, there was little difference in average concentrations of ^40^K among regions (Fig. S2).

### 3.2 Estimates of Daily Intake and Annual Committed Effective Dose of r-Cs and ^40^K

**Fig. 6.**
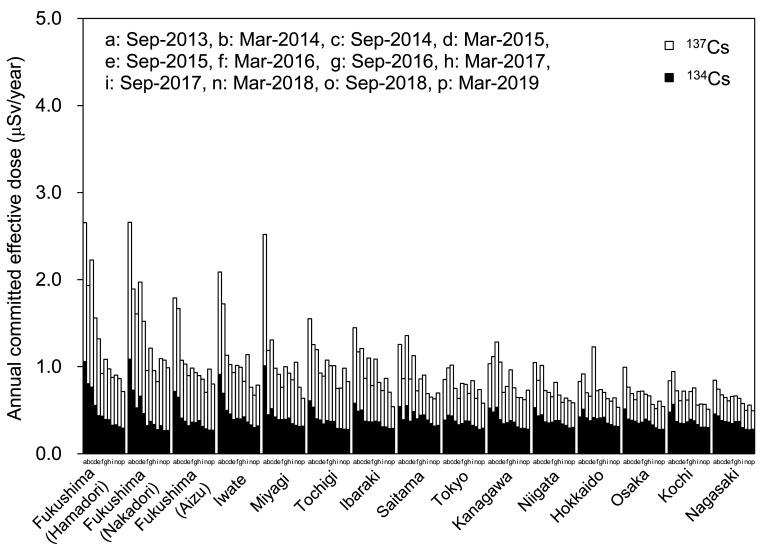
Time trends of annual committed effective doses of ^134^Cs and ^137^Cs in each region. Filled column and opened column indicate the annual committed effective doses of ^134^Cs and ^137^Cs, respectively. For each region, data are sorted according to the time of study.

We estimated daily intake and annual committed effective dose of r-Cs (^134^Cs and ^137^Cs) and ^40^K in the 15 regions (Tables 2 and 3, respectively). As described earlier, if the concentration of a given radionuclide in a MB sample was less than the LOD, half of the LOD was used as the concentration for calculation purposes. Therefore, it is necessary to consider the possibility that daily intakes and annual committed effective doses of r-Cs, especially those for ^134^Cs, were likely to have been overestimated in this study. The range of estimated daily intake and annual committed effective dose in each period is summarized in Tables 4A and 4B, respectively. Of note, the ranges were calculated only from quantifiable values, which means that not detected (ND) was handled as 0. Time trends for annual committed effective doses of ^134^Cs and ^137^Cs in the near-FDNPP region appeared to decrease from Sep-2013 to Mar-2019, however, the rate decrease was more gradual since Mar-2017 (Fig. 6). The daily intake and annual committed effective dose of r-Cs were at maximum in Fukushima (Nakadori) and Fukushima (Hamadori) before Sep-2015. However, the region, which had the maximum daily intake and annual committed effective dose of r-Cs, changed to other regions (Tochigi and Iwate) between Mar-2016 and Sep-2017. After Mar-2018, Fukushima (Nakadori) again became the region in which daily intake and annual committed effective dose of r-Cs were at maximum. There were no substantial regional differences in daily intake and annual committed effective dose from ^134^Cs in each year after Sep-2015 because ^134^Cs was not detected in almost all MB samples. Daily intake and annual committed effective dose of ^134^Cs in all regions slightly decreased in Sep-2017 and have since remained mostly stable, although the detection rate for ^134^Cs was already near zero in all regions before Sep-2017. This was probably caused by the lower LODs related to changes in the measurement environment, as described in the Materials and Methods section. Assuming that ^134^Cs is continuously not detected in any region in the future, it is expected that estimated daily intake and annual committed effective dose of ^134^Cs will be at similar levels, approximately 0.04 Bq/day and 0.3 μSv/year, respectively, given their respective LODs which have been at similar levels since Sep-2017. On the other hand, daily intake and annual committed effective dose of ^137^Cs in regions relatively close to the FDNPP such as Fukushima, Iwate, and Tochigi are still slightly higher than those in the far-FDNPP region. These results suggest that differences in daily intake and annual committed effective dose among regions were mainly attributed to ^137^Cs, which tended to decrease in the near-FDNPP region. Similarly, daily intake and annual committed effective dose of ^137^Cs in the far-FDNPP region appeared to decrease slightly, however, it was difficult to describe the time trend of ^137^Cs in these regions due to low detection rates and to changes in LOD during the study. Since detection rates for ^137^Cs in the far-FDNPP region were quite low (7~14%) and their concentrations were also low in Mar-2019, daily intake and annual committed effective dose of ^137^Cs were close to their respective LOD values, which were approximately 0.04 Bq/day and 0.2 μSv/year. As expected, the amount of daily intake and annual committed effective dose of r-Cs in the far-FDNPP region in our study were similar to the most recent values, approximately 0.08 Bq/day and 0.5 μSv/year, respectively, even if the detection rate for ^137^Cs has become 0%.

**Table 2.**
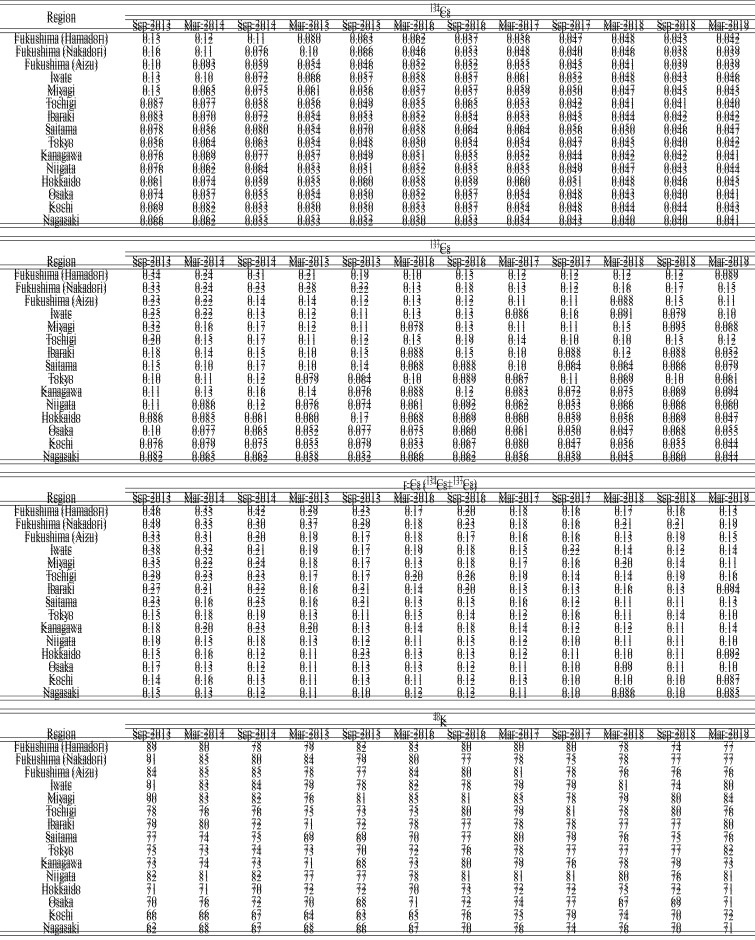
Daily intakes (Bq/day) of ^134^Cs, ^137^Cs, r-Cs, and ^40^K in 15 regions

These values were calculated using half of the LODs as concentrations of MB samples less than LODs.

**Table 3.**
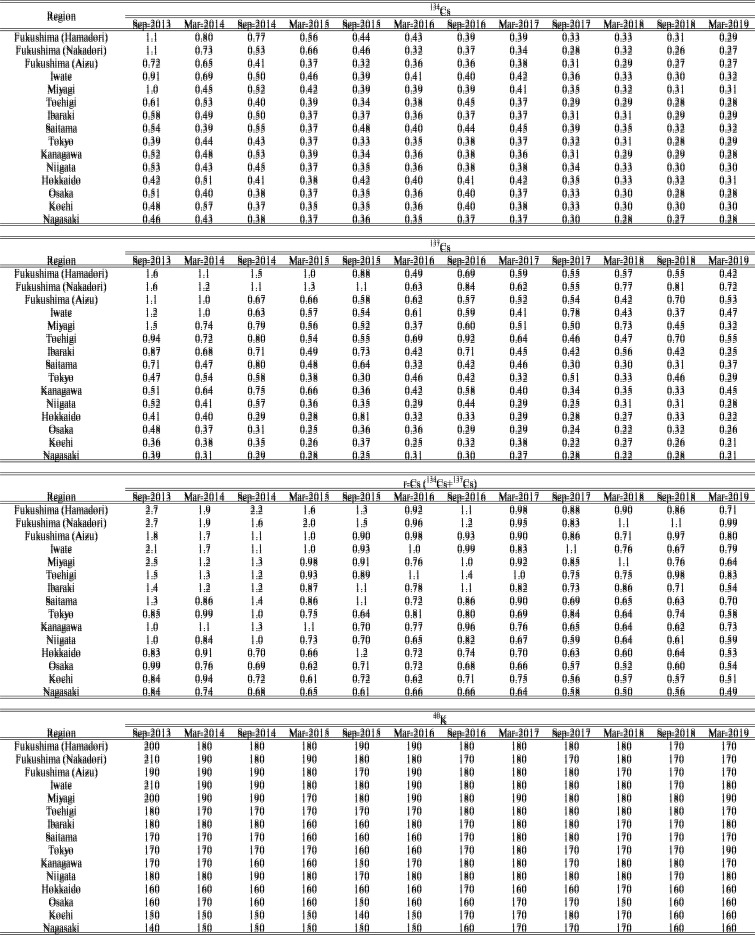
Annual committed effective doses (μSv/year) of ^134^Cs, ^137^Cs, r-Cs, and ^40^K in 15 regions

These values were calculated using half of the LODs as concentrations of MB samples less than LODs.

**Table 4.**
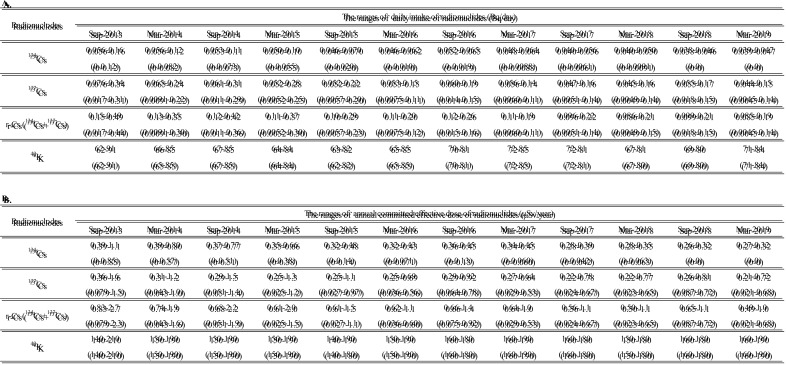
The ranges of daily intake (A) and annual committed effective dose (B) of radionuclides in each period

These values were calculated using half of the LODs as concentrations of MB samples less than LODs.

Shown in parentheses are the values calculated from only the quantifiable values, that means that ND were handled as 0.

Regarding food categories, because r-Cs were not detected or their concentrations were at very low levels, food consumption mainly contributed to the daily intake of r-Cs in the far-FDNPP region. Indeed, daily intake of r-Cs in category 9, which had the highest consumption, was estimated to be the highest among 14 food categories. Since the detection rate for ^137^Cs in categories 1, 2, 8, 10, and 12 was high in the near-FDNPP region, and food consumption of these categories was relatively high, daily intake of ^137^Cs in the near-FDNPP region was higher for these categories than for category 9. On the other hand, although a relatively high concentration of ^137^Cs was detected in category 5 in regions such as Iwate, Fukushima (Hamadori), and Fukushima (Nakadori), daily intake of ^137^Cs in this category was not high because the corresponding food consumption was low.

**Fig. 7.**
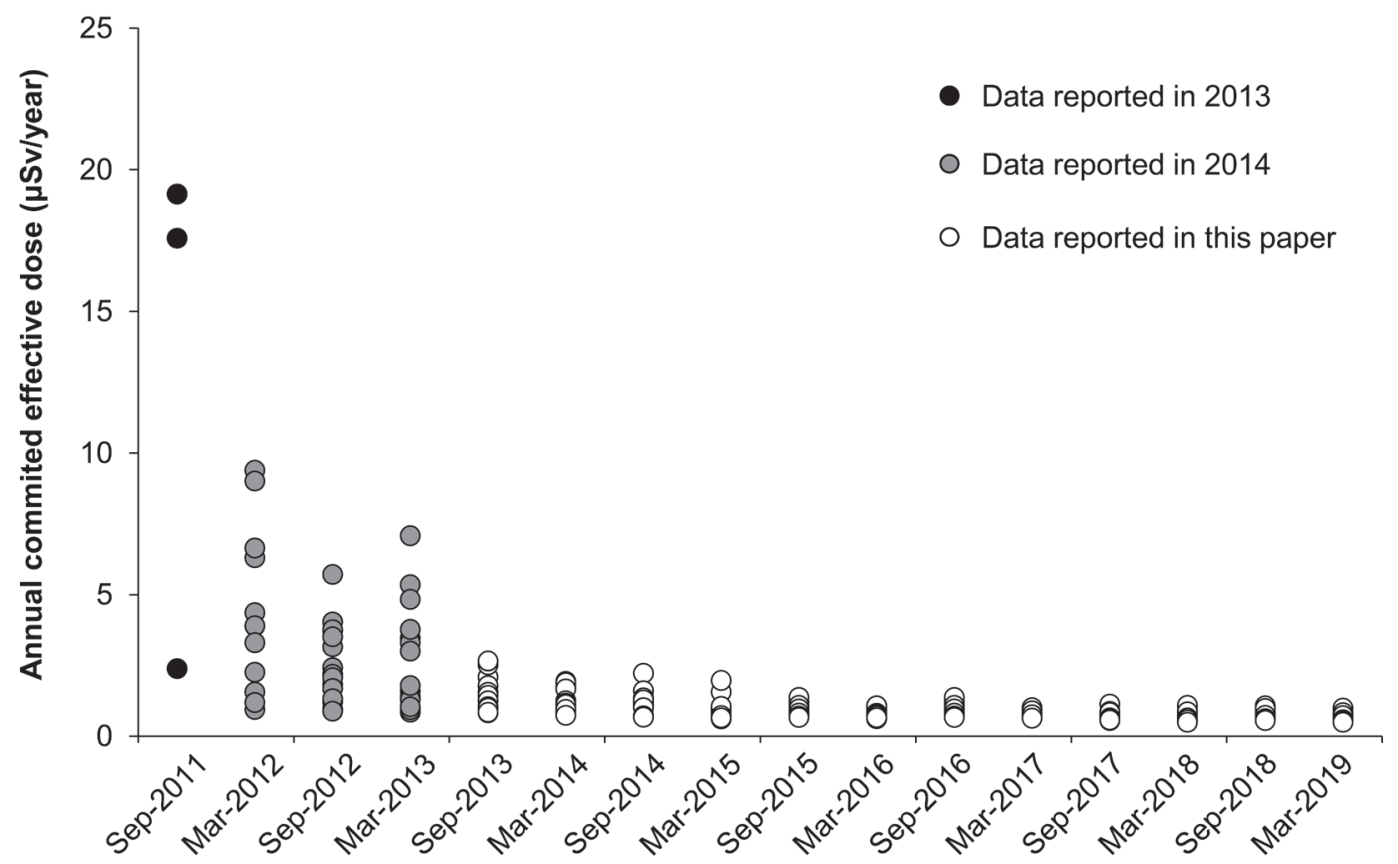
Time trends of the ranges of annual committed effective dose of r-Cs in this study and our previous reports. Filled circles and gray circles indicate previous data reported in 2013 and 2014, respectively. Opened circles indicate data in this study.

The ranges of annual committed effective dose of r-Cs among regions in this study were narrow compared with our previous study reported in 2013^17^^)^ and 2014^18^^)^ (Fig. 7). Compared with results before Mar-2013, the decreasing rate of annual committed effective dose of r-Cs during the 5.5-year duration of the present study was slow and was likely related to (1) an attenuation of ^134^Cs and ^137^Cs by their physical half-lives, (2) a decrease of r-Cs concentration in foods by production management, and (3) a decrease of r-Cs concentration in commercial domestic foods in Japan by the strict standard limit implemented in April 2012. We suggest that a drastic decrease in r-Cs concentration by the effects of countermeasures such as (2) and (3) largely contributed to a remarkable decrease in annual committed effective dose before Mar-2013. Since Sep-2013, the decrease in r-Cs concentration in distributed foods was thought to be slow, not drastic. Indeed, according to a report that summarized monitoring data released by the Ministry of Health, Labour and Welfare of Japan^26^^)^, r-Cs concentration in leafy vegetables, beef, cultivated mushrooms, and fishery products remarkably decreased until around 2013, after which it has slowly decreased or remained substantially unchanged.

Daily intake of ^137^Cs in Japan before the FDNPP accident was reported by Sugiyama et al in 2007^23^^)^. According to previous data, daily intake of ^137^Cs in 13 cities in Japan (Sapporo, Sendai, Saitama, Chiba, Yokohama, Niigata, Nagoya, Osaka, Kobe, Yamaguchi, Takamatsu, Fukuoka, and Naha) ranged from 0.012 to 0.042 Bq/day (calculated from only quantifiable values, which means that ND was handled as 0) and <0.038 to <0.080 Bq/day (calculated by assuming that concentrations less than the LODs were equal to the LODs, which means that ND was handled as the LOD), respectively. If these data were calculated by the present method (ND handled as half of the LOD), the daily intake of ^137^Cs would range from 0.027 to 0.055 Bq/day. Another study of daily intake of ^137^Cs by a food-duplicate survey was reported in the “environmental radiation database” by the nuclear regulatory agency of Japan^27^^)^. According to this database, daily intake of ^137^Cs excluding non-detectable data for the periods between 1999 and 2008 (the latest decade before the FDNPP accident) ranged from 0.0071 to 0.56 Bq/day. Since food-duplicate surveys appear to be affected by individual differences in food consumption habits, the range of daily ^137^Cs intake in that survey was considered to be large; median and 95th percentile were 0.028 and 0.063 Bq/day, respectively. On the other hand, the present results for daily ^137^Cs intake in Mar-2019 ranged from 0.052 to 0.15 Bq/day in the near-FDNPP region and from 0.044 to 0.060 Bq/day in the far-FDNPP region. Although a direct comparison of these results should be done cautiously because of differences in factors such as LODs in the analytical methods and ND assumptions, the most recent daily ^137^Cs intake in the far-FDNPP region was very close to or below the upper limits before the accident. On the other hand, the most recent daily ^137^Cs intake in parts of the near-FNDPP region was about two times higher than the upper limits before the accident. However, the maximum annual committed effective dose of r-Cs estimated by our most recent study in March 2019 was 1.0 μSv/year, which corresponds to approximately 0.1% of the intervention exemption level (1 mSv/year), a value sufficiently lower than the intervention exemption level. This finding suggested that r-Cs in commercial foods have been well-controlled in Japan.

On the other hand, daily intake and annual committed effective dose of ^40^K ranged from approximately 60 to 90 Bq/day and 150 to 200 μSv/year, respectively (Tables 2 and3), values that did not change much regardless of the time periods and regions. As an additional comparison, the dose received from ^40^K is estimated to be 165 μSv/year for adults, which is held fairly constant due to homeostasis^28^^)^. Compared with the maximum annual committed effective dose of r-Cs estimated in the present study, the maximum annual committed effective dose of ^40^K was approximately 75-fold higher. Furthermore, the annual committed effective dose of r-Cs was sufficiently lower than that for ^40^K (less than 2%) and was within the range of variance. This result indicated that humans usually intake the natural radionuclide, ^40^K, much more readily than they intake r-Cs.

We conclude that estimated daily intake and annual committed effective dose of r-Cs during the periods from Sep-2013 to Mar-2019 appeared to decrease gradually in the near-FDNPP region, and estimated annual committed effective doses of r-Cs were much lower than the intervention exemption level (1 mSv/year) in all regions and time periods. These results demonstrate that the health risk of ingestion r-Cs is significantly low when consuming normal diets that include commercial foods on the Japanese market. Nevertheless, to ensure food safety from the viewpoint of risk communication, it would be necessary to continue to monitor and disclose the daily intake and annual committed effective dose of r-Cs.

## Supplementary materials
